# Virtual Pedagogy and Care: Systematic Review on Educational Innovation with Mobile Applications for the Development of Healthy Habits in the Adolescent Population

**DOI:** 10.3390/nu15183966

**Published:** 2023-09-14

**Authors:** Cristina Arana-Álvarez, David Gómez-Asencio, Francisco-Javier Gago-Valiente, Yeray Cabrera-Arana, María-de-los-Ángeles Merino-Godoy, Emilia Moreno-Sánchez

**Affiliations:** 1 Department of Pedagogy, Faculty of Education, Psychology and Sport Sciences, University of Huelva, 21007 Huelva, Spain; cristina.arana530@alu.uhu.es (C.A.-Á.); david.gomez419@alu.uhu.es (D.G.-A.); yeray.cabrera@alu.uhu.es (Y.C.-A.); emilia@uhu.es (E.M.-S.); 2 Department of Nursing, Faculty of Nursing, University of Huelva, 21007 Huelva, Spain; angeles.merino@denf.uhu.es; 3 Center for Research in Contemporary Thought and Innovation for Social Development (COIDESO), University of Huelva, 21007 Huelva, Spain

**Keywords:** pedagogy, educational innovation, health care, mobile applications, public health, health promotion, adolescence, spiral curriculum

## Abstract

Background: The scientific literature was reviewed to determine the state of the art regarding innovative educational practices that employ mobile health applications with the aim of establishing healthy habits in the adolescent population, following a strategy based on spiral curriculum. Methods: The search was conducted in the WOS, Scopus and PubMed databases, discarding any articles that were not published in English, Spanish or French. The search was limited to studies performed in the last 6 years, that is, from 1 January 2017 to 18 March 2023. Results: From the initial sample of 132 articles selected, the final sample included 9 articles that met the eligibility criteria after a more exhaustive analysis. This systematic review identified one application (11.11%) with topics about sex and reproductive health, two applications (22.22%) that tackled mental health, six applications (66.66%) with topics about nutrition, three applications (33.33%) that contemplate physical activity, and two applications (22.22%) with aspects related to the prevention of harmful habits. The results show a positive relationship between the use of mobile health applications used as innovative didactic resources and the establishment of healthy habits in the adolescent population. Conclusions: Digital devices have been incorporated to the lives of humans as fundamental tools for their daily living. Specifically, adolescents are especially attracted to mobile phones. If this resource is used in this population to improve health, it is possible to attain very satisfactory objectives. The results of this review indicate that these devices can be used as a didactic resource in the promotion of health by implementing healthy habits at early ages, thereby contributing to the prevention of chronic diseases in future adulthood.

## 1. Introduction

Chronic diseases are the main cause of death and disability worldwide. At the global level, it is estimated that up to 80% of cases of heart diseases, strokes and type 2 diabetes, and over 30% of cases of cancer, can be prevented by reducing smoking and alcohol consumption, improving diet and practising physical activity regularly [1].

Disease prevention seeks to achieve the highest possible level of health in the population. However, aiming at improving health does not mean trying to reach immortality, and fighting for health is not equal to merely avoiding death. The aim is to fight certain types of deaths and to increase our life expectancy. The fact that we can live more years implies that, despite the increase in the probability of suffering from certain types of chronic–degenerative diseases that worsen our quality of life, we can delay their appearance, control them, know how to cope with them and, ultimately, add life to the years. In the family and school educational practice, the aim is to transmit applicable knowledge in their daily living and in their future as adults.

As basic knowledge taught from childhood, population health and lifestyles are addressed with their physical, chemical, biological and social components [2]. As was demonstrated by Jerome Bruner [3], the acquisition of knowledge, abilities and skills in childhood and adolescence consists in a spiral-learning method, where increasingly complex knowledge is progressively presented, integrating it by increasing the competencies related, in this case, to contents about healthy habits.

In the Ottawa Charter, the World Health Organisation [4] defines health promotion as the process that allows people to increase their control over determinants of health and, consequently, improve it. Similarly, they set requirements to reach good health, which depend on external determinants such as peace, education, housing, diet, decent work, healthy environment, social justice and equity, as well as on internal determinants such as beliefs, convictions, education, intelligence and emotions.

Health behaviours generally emerge during adolescence, persist in adulthood and commonly coexist [5,6,7]. Therefore, adolescence is a critical age to intervene and interrupt a trajectory toward poor adult health [8,9]. Physical inactivity, bad eating habits and practising harmful leisure activities are nowadays among the fundamental problems of the world society. Thus, it is necessary to generate some good health habits in the adolescent population, to ensure that they establish healthy behaviours and reach adulthood with good biopsychosocial health.

Moreover, this young population uses new technology very frequently, with smart phones becoming an integral part of their daily living. This circumstance can be used as an opportunity, since the use of mobile applications that foster healthy habits can be attractive and easy-to-access tools that transform learning into an innovative, creative, motivating and constructive process. To establish a habit in the population, a learning procedure is necessary. Mobile applications on healthy habits inherently carry a pedagogical process. In addition, since it is virtual learning, an innovative strategy is achieved [10].

For all of the above, the aim of this study was to carry out a systematic review of the scientific literature in order to identify intervention studies in adolescent populations that used mobile applications as an innovative didactic strategy to improve healthy habits, based on spiral curriculum. It is about investigating possible new health promotion strategies, in which new technologies are used for young people to establish healthy habits. If positive evidence is observed in this regard, personalized innovative health promotion strategies could be implemented.

## 2. Materials and Methods

In this study, a systematic review of the scientific literature was conducted, analysing studies with interventions aimed at promoting healthy habits through mobile applications in adolescent populations. The PRISMA criteria [11,12] for systematic reviews were applied, exclusively exploring the selected articles.

### 2.1. Selection Criteria

This systematic review was conducted in the months of February and March 2023 in the Web of Science (WOS), SCOPUS and PubMed databases, discarding articles that were not published in English, Spanish or French. The search was limited to studies performed in the last 6 years, that is, from 1 January 2017 to 18 March 2023, selecting open-access articles only.

### 2.2. Search Strategy

Regarding the search strategy, the following search criteria were selected: for WOS (Abstract: aplicacionesmóviles* OR mobile apps* OR applications portables* OR applications mobiles* AND adolescents* OR teenagers* OR adolescents* AND salud* OR Health* OR santé* AND promoción de la salud* OR Health promotion* OR promotion de la santé* AND hábitossaludables* OR healthy habits* OR habitudes saines*); for SCOPUS (TITLE-ABS-KEY (aplicaciones y móviles* OR mobile and trapps* OR applications and portables*) AND TITLE-ABS-KEY (adolescents* OR teenagers* OR adolescents* AND salud* OR health* OR santé* AND promoción de la salud* OR Health promotion* OR promotion de la santé*) AND pubyear > 2017 AND inglés OR francés OR español); for PUBMED (aplicacionesmóviles* [Title/Abstract] OR mobile apps* [Title/Abstract] OR applications portables* [Title/Abstract] OR applications mobiles* [Title/Abstract]) AND (adolescents* [Title/Abstract] OR teenagers* [Title/Abstract] OR adolescents* [Title/Abstract]) AND (salud* [Title/Abstract] OR Health* [Title/Abstract] OR santé* [Title/Abstract]) AND (promoción de la salud* [Title/Abstract] OR Health promotion* [Title/Abstract] OR promotion de la santé* [Title/Abstract]).

### 2.3. Inclusion and Exclusion Criteria

The following inclusion criteria were used: (a) studies in which some type of intervention with mobile applications was used; (b) the mobile applications were related to the area of health sciences; (c) the articles were published in scientific journals; and (d) studies whose sample was constituted by adolescent populations. Therefore, only original articles were included, selecting those with open access.

Since one of the inclusion criteria was the existence of intervention in the studies, the following documents were excluded: reviews, letters to editors, commentaries, opinions, perspectives, guidelines and rules, cases or case series, and systematic reviews. The adaptation of the selected articles to the objective and inclusion criteria of this review, with the aim of increasing the reliability and safety of the process, was independently carried out by three of the authors of this investigation (C.A.-A.; F.-J.G.-V.; and E.M.-S.). After reviewing the title, abstract and keywords of the article, if there were doubts about the inclusion of some articles, the other two authors (D.G.-A. and Y.C.-A.) made the final decision on their inclusion or exclusion.

**Figure 1:**
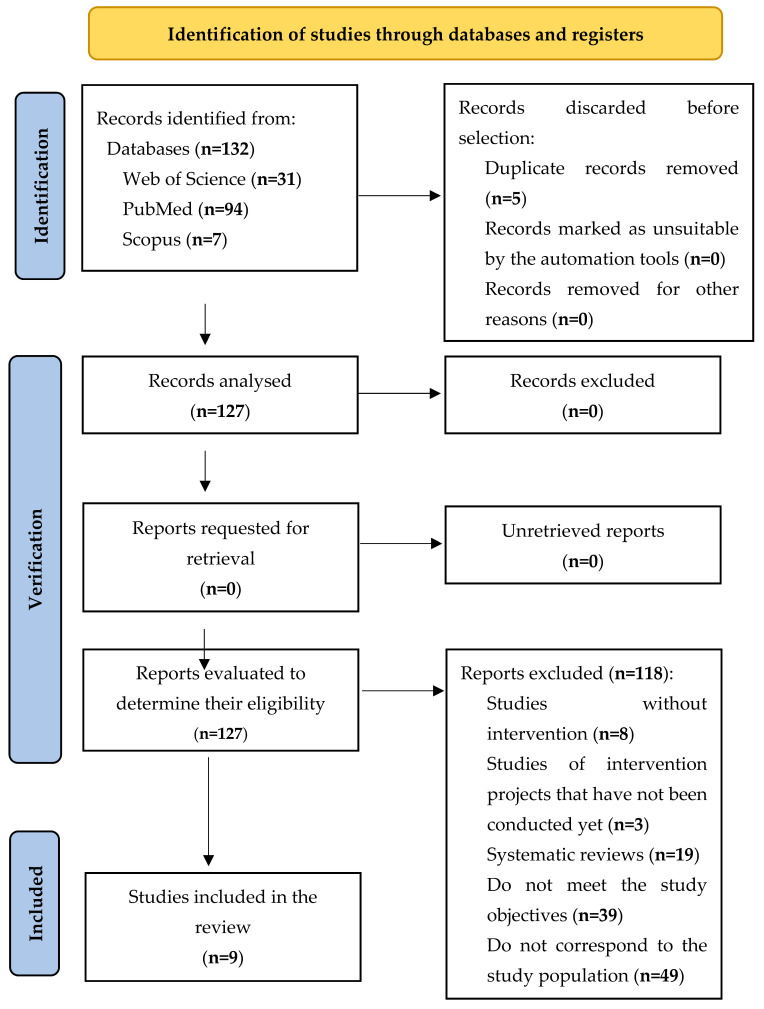
Flow chart of the systematic review process according to the declarations of the PRISMA protocol.

The identification and selection of articles (both included and excluded), and the reason for exclusion in the screening and selection phase, are shown in the flow chart of Figure 1.

### 2.4. Data Extraction

The data extraction process was carried out with numerous judgements and actions after the search. To this end, the first step was to exhaustively review the title, abstract, method, results and conclusions of each article. The data were extracted as they were published in their respective studies when they were reviewed.

This systematic review included variables following the PICOS acronym (P: participants; I: interventions; C: comparisons; O: outcomes; S: study design). Furthermore, this investigation included other variables that were considered relevant: authors, year of publication, country, reference of the article, study objectives, measurement variables, and scales.

### 2.5. Presentation of the Results: Adherence to Quality Initiatives (PRISMA)

The results of the primary studies, obtained through a systematic and reproducible methodology, are presented in a qualitative and quantitative manner (Figure 1).

### 2.6. Quality Evaluation

For the selection of the articles, a quality analysis was conducted following the quality evaluation components and scores of the EPHPP (Effective Public Health Practice Project) instrument [13]. With this instrument, a general assessment of each study was performed, evaluating six components (Table 1). Those studies without weak scores and at least four strong scores were considered strong. Those with less than four strong scores and one weak score were considered moderate. Lastly, the studies with two or more weak scores were considered weak [13].

The conclusions of this analysis are shown in Table 1. Of the nine articles included, four of them had a moderate global score [14,15,16,17], whereas the other five had a weak global score [18,19,20,21,22]. However, despite the fact that five of the articles presented global weak scores, they had strong internal components in variables such as data gathering and the percentage of participants who reached the end of the intervention. These internal components are relevant and can be prioritised with respect to other components since they are more strongly related to the study object of this systematic review. Therefore, although these five articles presented a global weak score, since their most relevant internal components were strong, they were included in this study. It is worth highlighting that six of the nine articles presented a strong score in the percentage of participants who reached the end of the interventions [14,16,17,18,19,22]. Another aspect worth pointing out is the use of validated instruments in most of the studies (60%) for data gathering, with strong scores in this component [14,15,17,18,19].

**Table 1:** Quality assessment components and ratings for the EPHPP instrument.

Articles	Components						
	1	2	3	4	5	6	Global Score
Steinberg et al. [18]	S	W	W	W	S	S	W
Hogsdal et al. [19]	M	W	S	W	S	S	W
Nagamitsu et al. [14]	M	S	S	W	S	S	M
Villasana et al. [20]	W	W	M	W	M	W	W
Caón et al. [15]	S	S	M	W	S	M	M
Thornton et al. [21]	S	W	M	W	M	M	W
Müssener et al. [22]	W	W	W	W	M	S	W
Lei et al. [16]	S	M	M	W	M	S	M
Vidmar et al. [17]	M	S	M	W	S	S	M

W = Weak; M = Moderate; S = Strong; 1 = Risk of bias; 2 = Design; 3 = Confounding factors; 4 = Masking; 5 = Data gathering; 6 = Withdrawals and dropouts.

## 3. Results

### 3.1. Selection of Studies and Data Extraction Process

After the search, the title, abstract and keywords of each article were reviewed with the aim of selecting those that could be relevant and discarding those that did not meet the inclusion criteria.

The searches were carried out and the inclusion criteria were applied, obtaining a total of 132 articles. The first search was performed in the Web of Science (WOS), where 31 articles were found; the second search was conducted in the Scopus database, gathering 7 articles; and the third search was carried out in PubMed, obtaining 94 articles.

Five articles were duplicates, and they were thus excluded, reducing the sample to 127 articles for full-text review. After applying the eligibility criteria, 118 articles were excluded, with 9 articles remaining in the final sample. The reasons for excluding 118 articles from the systematic review were that, although they initially met the inclusion criteria, after a more exhaustive reading, some of them were identified as systematic reviews (*n* = 19) or studies without intervention (*n* = 8), whereas others did not meet the study objective (*n* = 39), did not correspond to the study population (*n* = 49) or were studies in which intervention projects were proposed, but had not been carried out yet (*n* = 3). With the aim of reducing the selection bias, each article was reviewed independently by three of the researchers (C.A.-A.; F.-J.G.-V.; and E.M.-S.), who decided whether each document met the established criteria. In those cases in which these researchers did not reach consensus on the inclusion of an article, the other two researchers (D.G.-A. and Y.C.-A.) mediated the decision.

### 3.2. Characteristics of the Studies: Results Synthesis

Table 2 presents the following data of each of the studies: authors, year of publication, country, type of study, comparisons, study objectives, participants, variables and measurement instruments, interventions, and results. 

Of the nine studies included in this review, two (22.22%) were controlled randomised trials [17,21], one (11.11%) was an observational analytical study [18], one (11.11%) was a prospective, multi-institutional, controlled, randomised trial [14], three (33.33%) were cross-sectional analytical studies [15,19,22], one (11.11%) was an observational retrospective analysis [16], and one (11.11%) had a quasi-experimental longitudinal design [20].

Regarding the countries in which the different studies were conducted, two of the studies (22.22%) were carried out in the USA [17,18], one (11.11%) in Norway [19], one (11.11%) in Japan [14], one (11.11%) in Portugal [20], one (11.11%) in the UK, Italy and Spain [15], one (11.11%) in Australia [21], one (11.11%) in Sweden [22] and one (11.11%) in continental China [16].

In regard with the comparisons between groups in the analysed articles, two studies (22.22%) used an experimental group and a control group [15,17], one (11.11%) used two experimental groups and one control group [14], one (11.11%) used two experimental groups [19], and five (55.55%) used only one experimental group [18,20,21,22].

In relation to the study population in terms of sex, all studies (100%) analysed both men and women [14,15,17,18,19,20,21,22].

All the analysed studies conducted at least one type of intervention through some mobile application. These applications were: Teens in NYC [18], Opp and NettOpp [19], CBT (cognitive behavioural therapy) [14], CoviHealth [20], e-Diary Pegaso [15], Health4Life [21], LIFE4YOUth [22], MetaWell [16] and W8Loss2Go© [17].

With respect to the scopes addressed through the applications and in relation to health promotion, this systematic review identified one application (11.11%) with topics about sex and reproductive health [18], two applications (22.22%) that tackled mental health [14,19], six applications (66.66%) with topics about nutrition [15,16,17,20,21,22], three applications (33.33%) that contemplate physical activity [20,21,22], and two applications (22.22%) with aspects related to the prevention of harmful habits [21,22].

Of the nine articles analysed, eight (88.88%) of them concluded that the health applications used in their studies were useful for learning and improving health habits in the adolescents [14,15,16,17,18,19,20,21], and one (11.11%) demonstrated that the application used did not offer optimal functions and that it was fundamental to optimise the usability of the mobile health interventions in it [22].

### 3.3. Association between the Different Mobile Applications with Didactic Strategies for the Promotion of Healthy Habits

It was observed that the application used by one of the analysed studies offered information about providers of sex health services and contraceptive methods [18]. Two studies described applications with different didactic resources for the improvement of mental health, which included strategies to cope with stress and difficult situations [19] and skills to improve depressive symptoms, self-esteem, quality of life, self-control and health promotion in adolescents [14]. Six studies used applications that had resources to work on aspects related to nutrition, sport and the prevention of unhealthy habits; thus, the study of Villasana et al. [20] showed strategies aimed at monitoring, counselling and educating about nutrition and physical activity; Caón et al. [15] described resources for the proactive promotion of health (better eating behaviours: greater consumption of fruit and vegetables, and lower breakfast skipping); Thornton et al. [21] reported on activities in the application that were aimed at improving the six main health risk behaviours (bad diet, physical inactivity, smoking, alcohol consumption, sedentary recreational screen time and lack of or excess sleep); Müssener et al. [22] contemplated resources to promote healthy eating, physical activity, quitting smoking and reducing alcohol consumption; Lei et al. [16] identified as useful the application to facilitate weight loss in overweight and obese adolescents in the short and medium term, as well as to promote the practice of physical activity; and, lastly, Vidmar et al. [17] contemplated a series of activities to lose weight through physical training.

## 4. Discussion

The aim of this systematic review was to identify mobile applications that are used as an educational resource in health-related behaviours in adolescent populations.

The scientific literature shows that mobile applications can be useful and beneficial in the management and treatment of mental diseases, in combination with conventional therapy [23]. These applications represent new health promotion strategies with innovative and educational methodologies for the population. In this line, Pérez Márquez et al. [24] stated that the development of apps to favour mental health can be considered to be innovative ways of approaching the adolescent population. These findings are in line with the results of the present review, which shows outcomes of different applications that innovatively promote mental health through mobile applications that have been positively evaluated by the users [19]. However, these findings are not reported in similar studies that do not support the effectiveness of applications through goal attainment [25]. Education, self-control and goal setting are common components in the interventions of behavioural change designed for adolescents [26].

Nagamitsu et al. [14] showed that a well-care visit (WCV) with an interview of risk evaluation, counselling and self-control, and a mobile application of behavioural cognitive therapy (BCT/CBT) was more effective than receiving only WCV. The results were demonstrated by the attainment of self-control and depressive symptoms reduction skills. This finding shows the rapid acquisition of skills in this population, establishing the idea that health promotion during adolescence contributes to preventing the later appearance of mental health diseases, since the good habits are established at these ages are very likely to persist in adulthood [27].

Roldán [28] highlighted the need to use sex health apps not only in society, but also in the educational scope, both for adolescent students and for teachers and parents, since the latter would reduce their refusal or embarrassment toward addressing this topic. The review demonstrated a mobile application is an innovative way of covering important gaps in adolescent sex education and helping teachers to prepare their classes, ensuring that girls and boys understand everything. This would help to prevent and reduce the cases of STD (sexually transmitted diseases), sexual abuse and/or unwanted pregnancy. One of the studies analysed in this systematic review used a mobile application to promote sex and reproductive health among adolescents, obtaining promising results, which showed that the application helped the adolescents to discover and access a wide range of sex health services, including the least frequently used contraceptives [18].

Regarding the health education strategies related to nutrition and the promotion of active lifestyles, it is considered that the use of mobile applications is an effective resource for the acquisition of skills. Moreover, this helps to reduce long-term health risks [29]. This finding is reported in some of the studies analysed in this review, which conclude that health apps are useful for the promotion of healthy lifestyles, fostering the adoption of healthy eating habits, as well as good physical activity habits [20]; encouraging the change of behaviours and the improvement of eating habits [15]; indicating that they help to change life habits [21,22]; and pointing out their efficacy in weight loss among overweight adolescents [16,17]. According to several researchers, there are insufficient empirical data that determine the effect of motivation and of the use of apps in relation to some areas of the promotion of healthy habits, such as the practice of physical activity in adolescents [30].

There is an important prevalence in the perception of barriers to initiate or maintain physical activity habits, especially the lack of time or resources [31]. This variable could be palliated with the use of effective mobile health applications including motivating suggestions, dynamic advices and tips, gamification, challenges and the possibility to earn points [20], personalised suggestions, a virtual trainer, challenges and badges [15], rewards and notifications in the application to improve commitment [16,17,21], and personalised counselling [17], as was observed in many of the studies analysed in this systematic review.

Many of the studies analysed in this review conclude that an important strategy to promote the commitment and participation of the adolescent population in the use of mobile health applications is to involve them in the development of the latter [15,18,19,21]. The target population for the intervention should be involved in the design, aesthetics, usability and functionality [22]. The appearance of the application must be thoroughly worked on, since it must include an aesthetic value translated into colours, textures, sceneries, images, illustrations, icons, interactive widgets and effects that motivate adolescents, and it must also have a high functionality that leads to significant usability [32].

It must be taken into account that health apps pose a social advance and, a priori, an advantage in the world of health. They demonstrate the problem that most of the health applications that exist in the market are neither safe nor useful in terms of functionality. This difficulty can be palliated when there is professional supervision and satisfaction in terms of usability [33]. Many of the applications that have been pointed out in different studies of this systematic review have been analysed by experts and adolescents, presenting good results regarding safety and benefits [19,20,21,22].

With respect to the use of mobile health applications as a function of sex, it is demonstrated that, in one of the analysed studies, female adolescents presented more commitment with a mobile application to improve their diet and to have a healthy life compared to male adolescents [15]. This finding could be explained by the current information in the scientific literature, which shows that females choose to use more health-related applications, whereas males use more applications related to leisure and entertainment [34].

It is relevant to discuss limitations of the study, such as the relative scarcity of studies analysing the research question of this review. The search was only conducted in English, Spanish and French. In order to delve into the relationship between the use of mobile health applications in adolescent populations and the establishment of healthy habits, it would be interesting to perform more thorough systematic reviews, with more articles and languages, and with a meta-analysis. It would also be convenient to include more databases. Additional randomized controlled trials with different interventions and different mobile apps would improve our knowledge in this area.

## 5. Conclusions

It has been demonstrated there are different and varied applications focused on facilitating and/or improving health habits. The potential offered by this technology is very ambitious and can be used beneficially for the improvement of healthy habits in adolescent populations, as well as in the health education curriculum in the school and family scopes. The findings found in the nine articles reflect different health promotion strategies through new technologies, which is why educational innovation is evident.

The conclusion of this review is the positive relationship between the use of mobile health applications and the improvement of health habits. Their didactic use and contents with spiral structure enable their use in educational scopes, fostering cognitive, physical and social development and transforming educational organisations into health promotion spaces. Teaching healthy habits through new technologies represents a new approach to health promotion. More and more mobile applications are being developed for different fields. Specifically, those found in this review point towards a possible new perspective of teaching, a virtual pedagogy.

This study shows the usefulness of mobile health applications as a pedagogical tool for the establishment of healthy habits in adolescent populations, thereby contributing to the prevention of comorbidities in adulthood.

## Figures and Tables

**Table 2:** Characteristics of the studies included in the systematic review.

Author (Year) Country [Reference]	Study Design	Comparisons	Study Objectives	Participants	Measurement Variables (Scale Used)	Interventions	Results
Steinberg et al. (2018) USA [18]	Analytical, observational study	A single study group (the entire adolescent population of NYC that voluntarily downloaded the Teens app in NYC)	To promote sex and reproductive health among adolescents aged 12–19 years in New York	*n* = 22.137	Sex health services (birth control or gynaecological services, STD tests and treatment, pregnancy tests, abortion services, HIV tests, golden star service, mental health counselling and lesbian/gay/bisexual/transgender/queer (LGBTQ)–specific service), and different birth-control methods (condom, oral contraceptive pill, emergency contraceptives, injectable contraceptives, women’s condom, hormonal intrauterine device, copper intrauterine device, contraceptive implant, vaginal ring and birth-control patch)	This application has 3 main sections: Where to go, what to obtain and what to expect. Through different means, the application was disseminated among the study population, and the number of downloads was analysed, as well as the different sections used.	The results obtained in this study indicate that the application helped the adolescents to discover and access a wide range of sex health services, including the least used contraceptives. Therefore, the evaluation of the Teens mobile application in NYC suggests that mobile applications with search functionality are an effective way of providing information about sex health services to adolescents.
Hogsdal et al. (2023) Norway [19]	Cross-sectional study	Two groups: —One group of adolescents tried the Opp application (*n* = 27) —Another group of adolescents tried the NettOpp application (*n* = 15)	To investigate how adolescents evaluate the usability, quality and potential attainment of goals of Opp and NettOpp.	*n* = 42	Usability of the application (SUS scale), quality of the applications and attainment of the goals (knowledge, attitudes and intention to change the behaviour of the adolescents) (MARS scale).	The participants used the applications for a period of 3 weeks, and then they completed 2 cross-sectional questionnaires for users. The adolescents who tried Opp completed an online questionnaire, whereas those who tried NettOpp completed a paper questionnaire.	This study indicates that Opp and NettOpp have good usability and that the adolescents were satisfied with both applications. Most of the participants who evaluated Opp considered that the application would increase the user’s knowledge about mental health and help young people to cope with stress and difficult emotions and situations. Most of the participants who evaluated NettOpp stated that the application would raise awareness and knowledge about cyberbullying, change the attitudes toward cyberbullying and motivate them to address cyberbullying.
Nagamitsu et al. (2022) Japan [14]	Controlled, randomised, multi-institutional, prospective trial	-Two intervention groups: 1. WCV group (*n* = 66) 2. WCV + CBT application group (*n* = 66) -One control group without intervention (*n* = 70)	To test the efficacy of two interventions of health promotion in adolescents: a well-care visit (WCV) with an interview for risk evaluation, counselling and self-control, with a behavioural cognitive therapy (BCT) application for smart phones with the CBT application.	*n* = 202	Depression (DSRS-C scale), adolescent health promotion behaviours (AHP-SF scale), positive and negative global attitudes toward oneself (RSES scale), health-related quality of life (PedsQL scale), suicidal ideation (PHQ-9 scale), emotional intelligence traits—Short form for adolescents (TEIQue-ASF scale).	The participants of all groups were asked to complete a questionnaire that included several outcome measures in four time points: at the beginning and 1, 2 and 4 months after the beginning. For the group that only received the intervention with WCV, clinical data were gathered, and a physical examination was conducted in the first visit, and they received educational leaflets about health promotion aspects. In the next visits to the research centre, they completed an interview, and training sessions were also carried out. For the group that received the intervention with CBT, a programme was applied with a session of psychoeducation and another session of self-control. In this group, the participants had 10 different scenarios in the mobile application that taught different aspect about how feelings are associated with thoughts and actions.	There was significantly less suicidal ideation in the intervention groups. There was also a significant increase in the scores of the health promotion scale at 4 months of follow up among the secondary education students in the WCV group. Moreover, the CBT application was significantly effective in terms of obtaining self-control skills and reducing depression.
Villasana et al. (2020) Portugal [20]	Longitudinal, quasi-experimental design	The intervention was conducted in an experimental group, without a control group.	This study analysed the evolution of physical activity and nutrition habits of the adolescents involved in the study with a mobile application.	*n* = 7	Habits related to physical activity, food consumption and level of physical activity throughout the intervention (information gathering through questionnaires developed by the author).	The participants used the mobile application for five weeks, during which they were given 18 curiosities and 10 suggestions about nutrition and physical activity, as well as six challenges about the number of steps they had to take and calories that they had to burn. The mobile application was also used to administer four questionnaires related to the available advice and curiosities. During the study, the participants responded to four weekly questionnaires about the advice and curiosities that are provided by the mobile application.	The study demonstrated that a mobile application could be a complement for the promotion of healthy lifestyles, fostering the adoption of a healthy diet, as well as good physical activity habits.
Caón et al. (2022) England (UK), Scotland (UK), Lombardía (Italy) and Catalonia (Spain) [15]	Cross-sectional, analytical study	—One intervention group (*n* = 357) —One control group (*n* = 193)	To describe the methodology to implement the mobile register of foods of e-Diary Pegaso, evaluate its capacity to promote healthy eating habits, and assess the factors associated with its use and commitment.	*n* = 550	Evaluation based on diet and 6 target eating behaviours (DTB) related to the consumption of: fruit; vegetables; breakfast; sugary drinks; fast food; and sandwiches, continuous frequency of use in weeks (KIDMED), BMI, self-perceived health state (SPHS), socioeconomic level (FAS) and motivation to use the application (PCS).	The study scenarios selected were 12 secondary schools of four European places. A mobile phone was given with an application to provide proactive health promotion to all participants. For 6 months, the electronic diary followed-up on the eating habits of the users.	It was observed that, in those participants who were strongly committed to the e-Diary Pegaso application, there was an association with better eating behaviours: greater consumption of fruits and vegetables and lower breakfast skipping.
Thornton et al. (2021) Australia [21]	Controlled, randomised trial	A single group	To describe the development, usability and acceptability of the Health4Life application, which is an application of self-control for smart phones aimed at adolescents.	*n* = 232	Demographic characteristics (age, sex and post code) and 6 main lifestyle behaviours (bad diet, physical inactivity, smoking, alcohol consumption, sedentary recreational screen time, and lack of or excess sleep)	The participants were granted access to the Health4Life application, in which they had to record their health behaviours. Additionally, they had to complete an online questionnaire that evaluated the application, in order to generate information about its usability and acceptability.	In general, the students gave a favourable score to the Health4Life application. They considered it to be highly acceptable and usable, and they believed that it had the potential to efficiently and effectively modify important risk factors for chronic diseases among young people.
Müssener et al. (2020) Sweden [22]	Cross-sectional study	A single intervention group	To investigate the usability of an intervention of mHealth (LIFE4YOUth).	*n* = 5	Heuristic evaluation (visibility of the system’s state; similarities between the system and the real world; control and freedom of the user; consistency and standards; prevention of errors; identification instead of recovery; flexibility and efficiency of use; aesthetic minimalist design; helping the users to recognise, diagnose and recover from mistakes; help and documentation). Usability tests (see home page and explain what you think about the design; Where can you find support to drink less alcohol?; Record how much you drink; How to search for information about physical exercise activities; How to find a physical exercise activity and its participation level?; Describe how to set a goal for eating habits; What information can you find in the “Risks” section within the Diet module? Is it adequately visible in general?; Give us your opinion on the information provided in the first page of the Smoking module; How can you know more about the benefits of quitting smoking?).	All participants were invited to a brief training session (approximately 45 min), which was conducted by a research assistant (CL), in order to be briefed on the fundamental principles of heuristic evaluation and learn to use heuristics to evaluate the intervention. The participants were sent a link to a high-fidelity prototype of the intervention, included in the home page of the real software, the menu page and the 4 intervention modules (alcohol consumption, smoking, physical activity and diet). Each participant was asked to identify usability problems independently in a certain protocol. The participants were asked to identify a problem, describe it, identify the relevant heuristics for the problem and give it a severity score.	The usability tests showed that the design (aesthetics and clarity) and content (quality and quantity) enabled the usability of the application. However, the deficient functionality was considered an important barrier. One of the five participants gave a low score to the LIFE4YOUth intervention, two of them gave it an average score, and the other two evaluated it as good, according to the System Usability Scale. The mHealth intervention did not offer optimal functions, thus it entails the risk that students may stop using it. To sum up, it is fundamental to optimise the usability of the mobile health interventions of this application, in order to be applied with more satisfactory results.
Lei et al. (2021) Continental China [16]	Observational, retrospective analysis	A single group	To evaluate the efficacy of a digital health platform for weight loss using a mobile application (MetaWell), a wireless scale and calorie restriction with nutritional supplements in adolescents.	*n* = 2825	Age, sex, weight, percentile and BMI	The participants used a remote weight-loss programme that combined the use of a mobile application “MetaWell”, frequent self-weighing and calorie restriction with meal replacements. The changes in body weight were evaluated at 42, 60, 90 and 120 days using different metrics, including absolute body weight, BMI and Z score of BMI.	This study shows that the digital, remote weight-loss programme is effective at facilitating weight loss in overweight and obese adolescents in the short and medium term, since the participants achieved a clinically significant weight loss through this programme. Moreover, greater weight loss was observed in the older participants, in those who weighed themselves more frequently, and in those who had high baseline BMI percentiles.
Vidmar et al. (2018) USA [17]	Controlled, randomised trial	Two groups: One experimental group, who received intervention with App (W8Loss2Go©)[17] (*n* = 18). One control group, who received intervention in a clinic with conventional treatments (*n* = 17)	To evaluate whether an intervention with a mobile application of healthy habits could be a feasible and effective approach for adolescent patients with problems related to bad eating habits.	*n* = 35	Sociodemographic variables (sex, age, race, ethnicity); addictive behaviour (YFAS-c); abstinence from “problematic foods”; removal of elaborate meals; reduction in the amount of food in the meals; height; weight; addictive eating habits	The control group had a multidisciplinary intervention with conventional treatments. The interventions were carried out in monthly visits to the clinic for a period of 6 months. The individualised objectives of behavioural change were aimed at healthy eating, physical activity, emotional well-being and family support, which were followed up in the subsequent monthly visits. Another experimental group received an intervention with the W8Loss2Go© application. The intervention included two visits to the clinic at 3 and 6 months (45 min per visit), text messages 5 days per week, and phone sessions every week (15 min × 24 sessions = 360 min). The intervention was focused on three characteristics of the addictive eating behaviour: (i) gradual removal of problematic foods identified by the participant; (ii) staggered removal of sandwiches between meals; and (iii) abstinence from excessive amounts of foods in the meals.	The group that received the intervention with the mHealth App “W8Loss2Go” [17] obtained better results in the improvement of BMI and in the effective completion of the therapeutic treatment, compared to the group that received the intervention in the clinic with conventional treatments.

## Data Availability

Further data that support the findings of this study are available upon reasonable request from the corresponding author. Some data are not publicly available due to privacy or ethical restrictions.
